# The role of the 5*f* valence orbitals of early actinides in chemical bonding

**DOI:** 10.1038/ncomms16053

**Published:** 2017-07-06

**Authors:** T. Vitova, I. Pidchenko, D. Fellhauer, P. S. Bagus, Y. Joly, T. Pruessmann, S. Bahl, E. Gonzalez-Robles, J. Rothe, M. Altmaier, M. A. Denecke, H. Geckeis

**Affiliations:** 1 Karlsruhe Institute of Technology (KIT), Institute for Nuclear Waste Disposal (INE), P.O. 3640, D-76021 Karlsruhe, Germany; 2 Department of Chemistry, University of North Texas, Denton, Texas 76203-5017, USA; 3 University Grenoble Alpes, Inst NEEL, F-38042 Grenoble, France; 4 CNRS, Inst NEEL, F-38042 Grenoble, France

## Abstract

One of the long standing debates in actinide chemistry is the level of localization and participation of the actinide 5*f* valence orbitals in covalent bonds across the actinide series. Here we illuminate the role of the 5*f* valence orbitals of uranium, neptunium and plutonium in chemical bonding using advanced spectroscopies: actinide M_4,5_ HR-XANES and 3*d*4*f* RIXS. Results reveal that the 5*f* orbitals are active in the chemical bonding for uranium and neptunium, shown by significant variations in the level of their localization evidenced in the spectra. In contrast, the 5*f* orbitals of plutonium appear localized and surprisingly insensitive to different bonding environments. We envisage that this report of using relative energy differences between the 5*f*δ/ϕ and 5*f*π*/5*f*σ* orbitals as a qualitative measure of overlap-driven actinyl bond covalency will spark activity, and extend to numerous applications of RIXS and HR-XANES to gain new insights into the electronic structures of the actinide elements.

## Abstract (web summary)

Understanding the chemistry of heavy radioactive elements is crucial to harnessing them for fuel and medicine. Here, the authors combine advanced X-ray spectroscopy and modelling to the study the chemical bonding of the 5*f* valence orbitals of uranium, neptunium and plutonium.

One of the long lasting controversial debates in actinide chemistry is the level of localization and participation of the actinide (An) 5*f* valence orbitals in covalent bonds across the actinide series^1^,^2^. The 5*f* orbitals of the lighter An elements can be very active up to Am; thereafter, they strongly contract and are considered as core-orbitals similar to their 4*f* counterparts, the lanthanides (Ln). Advanced spectroscopy methods can provide new insights into the electronic structure of the An elements and thereby help resolve this controversy, as well as promote development and optimization of theoretical approaches to predicting their chemical and physical properties. Progress in modern theory now permits precise electronic structure calculations of large An systems in an excited state relevant to advanced spectroscopy, where not only valence but also core-electrons are removed from the atom^2^,^3^,^4^. Marriage of advanced spectroscopy and theoretical modelling delivers mechanistic understanding of the electronic structure of An radioelements crucial to a number of applications. Those include the improved understanding of An interaction with the constituents of the geo and ecosphere on a molecular scale being highly relevant for the reliable assessment of their behaviour in the environment^5^,^6^,^7^,^8^,^9^. Understanding the electronic structure of An bondings is also of crucial importance for the design and optimization of selective and stable ligands for highly efficient separation processes for closed nuclear fuel cycle concepts and the potential application of actinides in nuclear medicine^10^,^11^,^12^.

We are applying An M_4,5_ absorption edge high-energy resolution XANES (HR-XANES; XANES=X-ray absorption near edge structure) and 3*d*4*f* resonant inelastic X-ray scattering (3*d*4*f* RIXS) as bulk sensitive techniques, which can be applied *in-situ* and under extreme conditions (for example, high/low temperatures or pressures) for investigations of matter in any state^13^,^14^,^15^,^16^,^17^,^18^,^19^. Particularly 3*d*4*f* RIXS is a novel experimental tool for comparison of the level of localization of unoccupied 5*f* orbitals across the An series and their participation in chemical bonding. In this report, we investigate uranium (U)/neptunium (Np)/plutonium (Pu) in different oxidation states in several materials in solid/liquid state. One focus is on U, Np and Pu in their hexavalent oxidation states U(VI), Np(VI) and Pu(VI) in aqueous 1 M perchloric acid (HClO_4_), where they form strong covalent trans dioxo linear bonds, also often referred to as actinyl (AnO_2_ ^2+^) bonding, with short axial oxygen, An-O_ax_, bonds. Five loosely bound equatorial water (H_2_O) molecules are located in the equatorial plane of the actinyl cation. Under oxidizing and aerobic conditions AnO_2_ ^2+^ can be quite stable where UO_2_ ^2+^ clearly has the highest stability and NpO_2_ ^2+^ might only be of relevance under hyperalkaline conditions. UO_2_ ^2+^ is frequently found in the environment as a constituent of mineral phases but also as a highly mobile aqueous species or in colloidal form^7^,^8^,^20^.

Experimental results are compared to and interpreted using theoretically calculated data. Pu(VI) M_5_ absorption edge HR-XANES is calculated with multiplet calculations using the DIRAC programme system^21^ and with the finite difference method (FDMNES code^22^). The object of these calculations is to verify if the An M_4,5_ absorption edge HR-XANES spectra are sensitive to the ligand field and if they closely describe the ground state 5*f* partial density of states (5*f*-DOS) of the An.

As well as the orbital model, other one-electron effects, in particular spin–orbit splittings, and many-body effects, in particular multiplet splittings, contribute in a major way to the XANES spectra. Furthermore, the relative importance of the ligand-field splittings and the spin–orbit splittings may need to be considered. These other effects and their contributions to the XANES spectra are discussed.

Here we provide insight into one of the most controversial questions in actinide chemistry: What is the role of the 5*f* valence orbitals in the covalency of the chemical bonding of the actinide elements? HR-XANES and RIXS at the M_4,5_ absorption edges of the early actinide elements (U, Np, Pu) probe the 5*f* unoccupied density of states of the actinides with superior energy resolution. These advanced spectroscopy tools allow to distinguish between increase of covalency due to building up of electronic charge between the atoms, classical overlap of orbitals, and due to better energy match between metal and ligand valence orbitals as illustrated for U(VI)O_2_ ^2+^, Np(VI)O_2_ ^2+^ and Pu(VI)O_2_ ^2+^. It is demonstrated that the 5*f* orbitals are more localized for Pu compared with U and Np, and their energies are not influenced by changes in bonding environments. A broad set of data obtained from different actinide compounds are compared—including spectra for aqueous and solid state species as well as highly radioactive materials like spent nuclear fuel. Spectroscopic results are corroborated by state of the art quantum-chemical calculations.

## Results

### Molecular orbital scheme of actinyl

**Figure 1:**
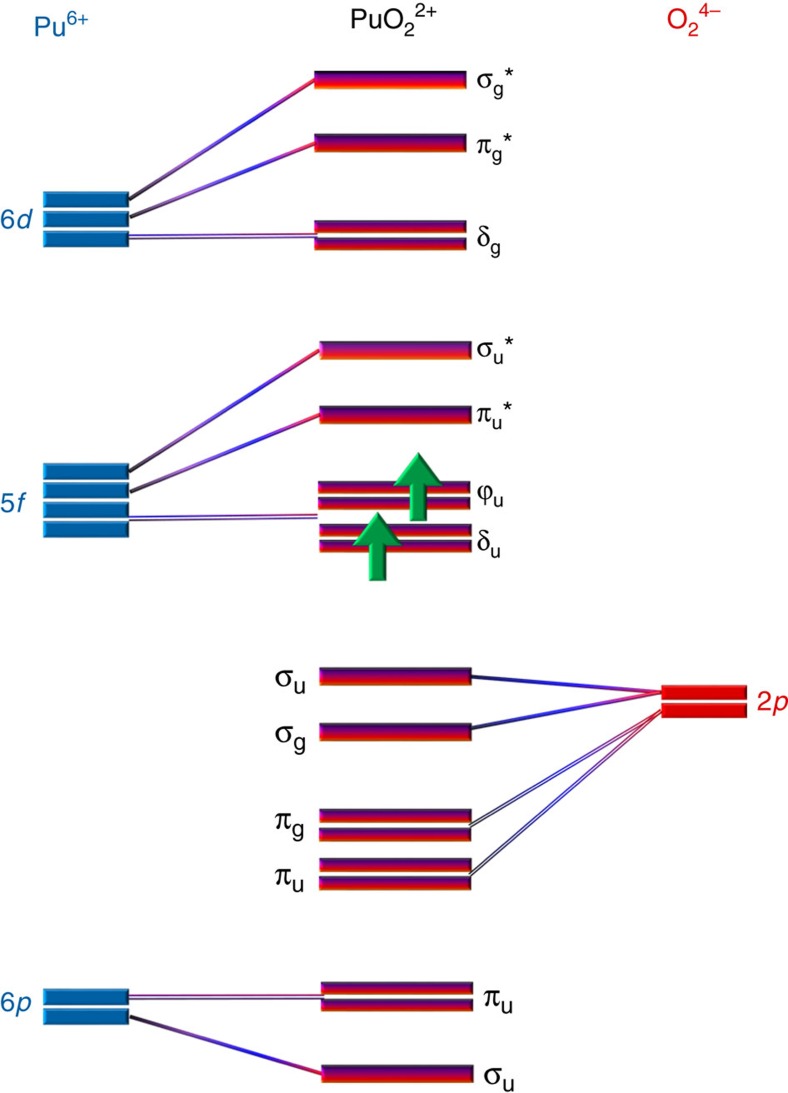
Molecular orbital scheme. A qualitative molecular orbital scheme of PuO_2_ ^2+^ adapted from (refs 23, 24). Only frontier electrons are indicated.

The An oxidation states and their electronic structures are interconnected^2^,^3^,^4^,^23^; a qualitative molecular orbital scheme of PuO_2_ ^2+^ is depicted in Fig. 1 (refs 23, 24). Note that in Fig. 1 the spin–orbit splitting of the 5*f* levels is not shown, An estimate of these splittings can be obtained from the data for UO_2_ in Fig. 1 of ref. 25, where the *5f*π_3/2_ to 5*f*π_1/2_ splitting is shown to be ∼1.2 eV. This splitting is significant for the broadening of features in the HR-XANES as discussed later. The linear structure of the AnO_2_ ^2+^ molecule (Supplementary Fig. 1) leads to the An valence orbitals being most usefully described in terms of their σ, π, δ and ϕ character with respect to rotation about the axial An-O_ax_ *z* axis (*D*_∞h_ point group symmetry). The σ and π are anti-bonding with the O 2*p* orbitals and are denoted σ* and π*, while the δ and ϕ are non-bonding for a gas phase molecule. The δ and ϕ might become π* and, σ* and π* with respect to weakly bound equatorial ligands. For simplicity, we use only the assignments σ, π, δ and ϕ, for the *D*_∞h_ symmetry of linear AnO_2_ ^2+^. An M_4,5_ absorption edge HR-XANES and 3*d*4*f* RIXS directly measure the relative energies of the unoccupied 5*f*δ/ϕ, 5*f*π* and 5*f*σ* orbitals of the An in the same spectrum, thus avoiding experimental artifacts introducing uncertainties due to different sample preparation protocols or variable measurement conditions^17^. Especially, the 5*f*σ* orbital can be evaluated with high precision. The relative energies of these lowest lying unoccupied orbitals of the An with mainly 5*f*-based character can also be measured with optical (5*f*δ, 5*f*ϕ, 5*f*π*) and X-ray absorption/emission spectroscopy (5*f*π*, 5*f*σ*), yielding excited state data that deviate <0.5 eV compared with the theoretically calculated ground state values^3^. However, these two methods have limitations, including low temperature requirement for precise optical spectroscopy measurements and ultra-high vacuum for, for example, O K absorption edge XANES^3^.

### Actinide 3*d*4*f* RIXS maps

**Figure 2:**
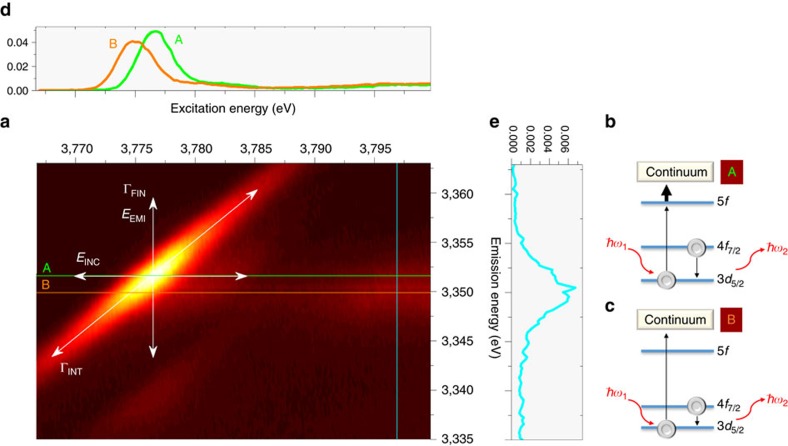
RIXS map of PuO_2_ and schematics of the emission process. (**a**) 3*d*4*f* RIXS map of PuO_2_. (**b**) One-electron scheme of the emission process for excitation energies of about 3,776.5 eV and (**c**) above 3,785 eV. The intense resonant structures at about 3,351.6 eV emission energy in the 3*d*4*f* RIXS map correspond to 4*f*_7/2_→3*d*_5/2_ Pu M_α_ emission, whereas the peaks with weaker intensity at about 3,340 eV emission energy describe 4*f*_5/2_→3*d*_5/2_ Pu M_α_ emission. The energy positions of the most intense resonance (3,351.6 eV) and the normal emission line (3,350 eV) for the 3*d*_5/2_→5*f* Pu M_5_ absorption edge and 4*f*_7/2_→3*d*_5/2_ Pu M_α_ emission, are marked with lines A and B, respectively; (**d**) the HR-XANES extracted for lines A and B; (**e**) the emission line measured at 3,797 eV excitation energy. The experimental broadening depending on the specifications of the beamline (*E*_INC_), and the spectrometer (*E*_EMI_), as well as the core-hole lifetime broadening effects in the intermediate (Γ_EMI_) 3*d*^9^5*f*^N+1^ (*N*=number of electrons in the An 3*d*^10^5*f*^N^ ground state) and final (Γ_FIN_) 4*f*^13^5*f*^N+1^ states, are indicated.

A RIXS map depicts a 2D representation of X-ray fluorescence emission measured as a function of the excitation X-ray energy scanned across an absorption edge (Fig. 2a)^13^,^26^,^27^,^28^,^29^. If the excited electron (for example, 3*d*_5/2_ of Pu, 3*d*_5/2_→5*f* Pu M_5_ absorption edge) and the electron filling the created core-hole (for example, 4*f*_7/2_ of Pu, 4*f*_7/2_→3*d*_5/2_ Pu M_α_ emission) are close to the nucleus, that is, core-electrons, the map is denoted as a 3*d*4*f* core-to-core RIXS map (3*d*4*f* CC-RIXS). For simplicity, we will refer to the 3*d*4*f* CC-RIXS maps recorded at the U M_4_ (3*d*_3/2_→5*f* excitation, 4*f*_5/2_→3*d*_3/2_ U M_β_ emission) and Pu/Np M_5_ absorption edges as RIXS. The RIXS map of PuO_2_ and a simplified one-electron scheme describing the emission process are shown in Fig. 2. The 3*d*_5/2_ electrons are mainly excited to the unoccupied states with substantial 5*f* character (3*d*_5/2_→5*f*); the orbital angular momentum quantum number *l* changes ±1, according to the dipole selection rule, Δ*l*=±1.

**Figure 3:**
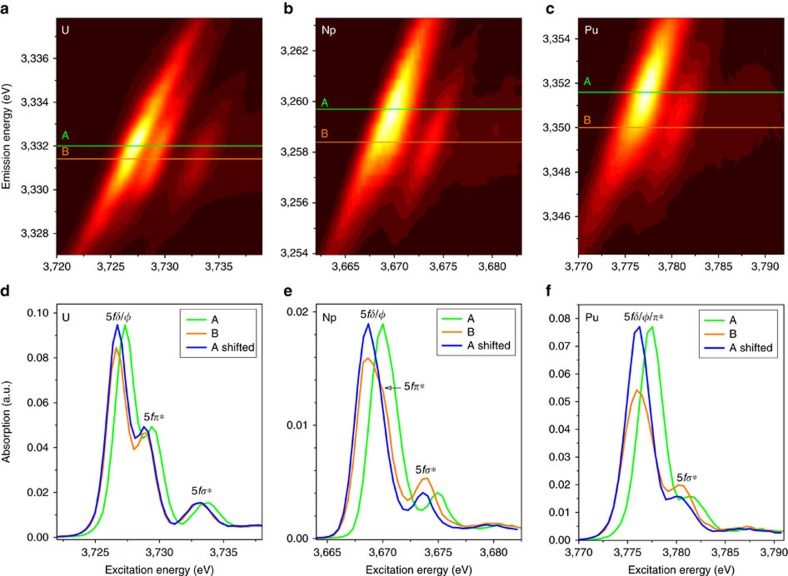
RIXS maps and extracted HR-XANES spectra. (**a–c**) 3*d*4*f* RIXS maps (3*d*_3/2_→5*f* U M_4_ absorption edge, 4*f*_5/2_→3*d*_3/2_ U M_β_ emission; 3*d*_5/2_→5*f* Np/Pu M_5_ absorption edge, 4*f*_7/2_→3*d*_5/2_ Np/Pu M_α_ emission) and (**d**,**e**) U M_4_ and Np/Pu M_5_ absorption edge HR-XANES spectra extracted along lines A and B for (**d**) UO_2_ ^2+^, (**e**) NpO_2_ ^2+^ and (**f**) PuO_2_ ^2+^.

**Figure 4:**
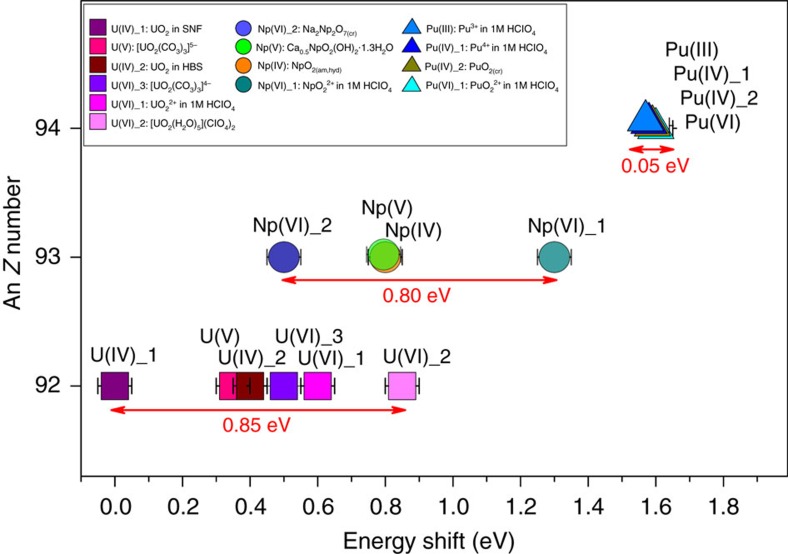
Energy shifts obtained from RIXS maps. Energy shift of the main resonance peak (line A in Figs 2 and 3) compared to the energy position of the normal emission line (line B in Figs 2 and 3) for different U, Np and Pu materials measured at the U M_4_ (filled squares) and Np/Pu M_5_ absorption edge (Np: filled circles and Pu: triangle). Spent nuclear fuel and high-burn up structure are abbreviated with SNF and HBS, respectively. Information about the samples in given in SI (cf. Supplementary Table 1; Supplementary Note 1).

The RIXS map can be divided into a resonant emission and a non-resonant (normal) emission, where the electron is excited to bound unoccupied states (<3,785 eV excitation energy) and to the continuum (>3,785 eV excitation energy), respectively, both with subsequent 4*f*_7/2_→3*d*_5/2_ Pu M_α_ emission. Simplified schemes of these two processes are depicted in Fig. 2b,c. The RIXS maps of UO_2_ ^2+^, NpO_2_ ^2+^ and PuO_2_ ^2+^ are shown in Fig. 3a–c. Typically when recording a HR-XANES spectrum, the analyzer crystals of an X-ray emission spectrometer are positioned at the energy position of the maximum of the normal emission line and the energy of the incident beam is scanned across the absorption edge^27^,^28^,^30^. Then the HR-XANES spectrum corresponds to a cross section across the RIXS map marked with line B in Figs 2 and 3. The broadening of the spectral peaks depends on experimental and core-hole broadening effects. The beamline (*E*_INC_) and the spectrometer (*E*_EMI_) experimental broadening contribute along the *x* and *y* axis of the RIXS map, respectively (Fig. 2). The smaller core-hole lifetime broadening^31^,^32^ in the final state (Γ_FIN_) mainly contributes to the HR-XANES spectrum (Fig. 2; Supplementary Table 1)^28^,^33^; in our case, Γ_FIN_ equals 4*f*^13^5*f*^N+1^, where *N* is the number of electrons in the ground state 3*d*^10^5*f*^N^ of the An (0, 1 and 2 for UO_2_ ^2+^, NpO_2_ ^2+^ and PuO_2_ ^2+^). The HR-XANES spectrum can exhibit different features compared with the conventional XANES spectrum when the core-hole potential in the intermediate (3*d*^9^5*f*^N+1^) state is significantly different than that for the final state (4*f*^13^5*f*^N+1^)^34^,^35^,^36^,^37^,^38^. This effect is well visible in Figs 2a and 3a–c, where line B marking the normal emission maximum is shifted with respect to the most intense resonant peak, line A. The HR-XANES spectra extracted at emission energy marked with line A are also shown in Figs 2d and 3d–f. Three peaks at about 3,726.7, 3,728.8 and 3,732.9 eV are resolved in the UO_2_ ^2+^ RIXS map, which are also visible in the HR-XANES spectra (Fig. 3d). We have previously been able to assign these to transitions of 3*d* electrons to 5*f*δ/ϕ, 5*f*π* and 5*f*σ* orbitals, respectively^17^. The first two peaks 5*f*δ/ϕ and 5*f*π* are not resolved in the NpO_2_ ^2+^ and PuO_2_ ^2+^ RIXS maps/HR-XANES, but a peak shoulder (indicated with an arrow and 5*f*π*) and asymmetry are evident for NpO_2_ ^2+^ (Fig. 3d,e). To better compare spectra, we removed the energy shift between A and B spectra so they appear overlaid. In the overlaid spectra, features have similar energy positions but the main peak intensities for the A spectra are much higher than the B (normal emission) spectra. Note that the shoulder observed on the main peak in the NpO_2_ ^2+^ B spectrum is not visible in the A spectrum. When the entire emission intensity is integrated symmetrically with respect to the normal emission line (line B), the resulting XANES spectrum corresponds to a conventional fluorescence mode measurement (Supplementary Fig. 2b). The conventional spectrum is dominated by one broad absorption feature, without a shoulder, but with a maximum shifted to higher energies than the B HR-XANES spectrum due to the contribution of the lower energy intense resonance marked with line A (Fig. 3a–c). The energy difference or shift between lines A and B is 0.6, 1.3 and 1.6 eV for UO_2_ ^2+^, NpO_2_ ^2+^ and PuO_2_ ^2+^ in 1 M HClO_4_, respectively. These values are compared with those measured for other materials in Fig. 4. The size of this shift varies between 0–0.85 eV (U) and 0.5–1.3 eV (Np) for the various materials, whereas it remains the same for Pu(III)/Pu(IV)/PuO_2_^2+^ in 1 M HClO_4_ and PuO_2_. Emission energy shift of the resonant peaks with respect to the normal emission was previously reported for 3*d*^36^ and 4*f*^34^,^35^,^37^,^38^ elements and was attributed to the strong interaction between the excited electron in the lowest unoccupied bound electronic states and the created core-hole, which differs from the ionized case, that is, excitation into continuum. The energy shift between lines A and B, therefore, probes the localization of those lowest unoccupied 5*f* states on the absorbing atom. Schematics of the effect are shown in Fig. 2b,c. The experimentally observed increase in energy shift going from U to Pu in our actinyl system shows that the 5*f* orbitals undergo stronger localization in this order, that is, from UO_2_ ^2+^ to NpO_2_ ^2+^ than from NpO_2_ ^2+^ to PuO_2_ ^2+^. Note that the shift can be slightly different for the U M_5_ absorption edge RIXS due to associated variations in the core–electron interaction. Observed invariance of the Pu energy shift indicates that the localization of the Pu 5*f* orbitals is not influenced by changes in oxidation state and appears to be the same for molecular and solid state species. This is not true for U and Np, as significant variations are observed for these elements. An important observation is also that the main resonance peak (line A) is shifted to higher energies with respect to the normal emission (line B), a trend opposite to pre-edge peaks in K edge RIXS measurements for transition metals^36^. This effect might be related to the relativistic nature of the core-electrons of the An elements, yielding strong contraction of the electronic orbitals for the ionized atoms.

### Actinide M_4,5_ absorption edge HR-XANES

**Figure 5:**
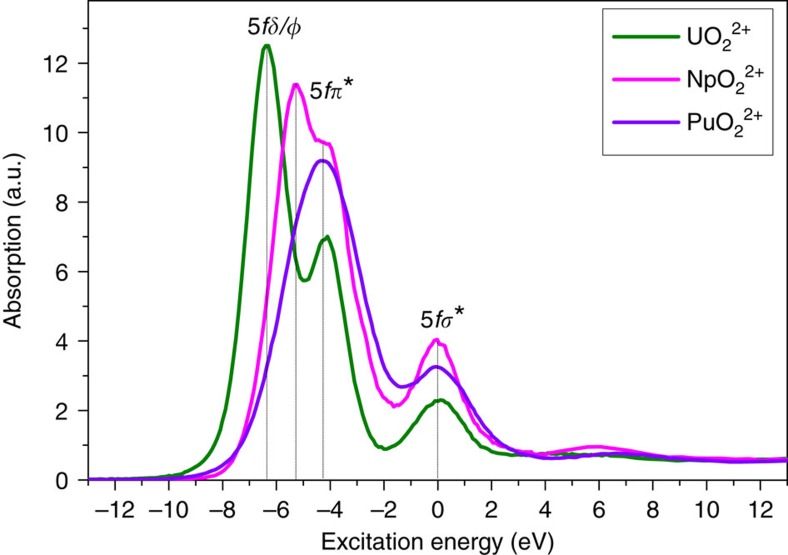
Experimental HR-XANES spectra. U M_4_ and Np/Pu M_5_ absorption edge HR-XANES spectra of UO_2_ ^2+^, NpO_2_ ^2+^ and PuO_2_ ^2+^.

In Fig. 5 the HR-XANES spectra of UO_2_ ^2+^, NpO_2_ ^2+^ and PuO_2_ ^2+^ are aligned so that the 5*f*σ* peak is at 0 eV. Comparison of the HR-XANES reveals differences in intensity and energy position of resonant features. The main 5*f*δ/ϕ peak intensity changes drastically going from U to Pu, since the non-bonding orbitals are free for U but have *5f*δ^1^ and 5*f*δ^1^5*f*ϕ^1^ configurations for Np and Pu, respectively. In addition, changes in the intensity can be linked to the increasing energy shift between the most intense emission resonance (line A) and the normal emission (line B). For many of the An materials the first 5*f*δ/ϕ peak in the HR-XANES is associated with a cross section of the tail of the emission spectrum and not the maximum of the resonance peak of the RIXS spectra (cf. B spectra in Figs 2 and 3). The multiplet splittings with highest importance for Pu(VI) also introduce broadening, yielding decreased peak heights (cf. calculations of Pu(VI) M_5_ HR-XANES spectra below). As the An(VI) samples are in aqueous solutions and their concentrations are the same, we rule out self-absorption effects as playing any substantial role in damping of intensities of spectral features.

The energy difference between features hints at differences between bonding covalencies. The seven empty or partially occupied 5*f* orbitals of the AnO_2_ ^2+^ cation (Fig. 1) are split by spin–orbit coupling and the ligand field of the axial and equatorial ligands (ax. field and eq. field). When there is more than one electron in the 5*f* shell, Coulomb repulsions between the electrons become important and affect the covalent mixing^39^. The spin–orbit splitting of the 5*f* electrons is ∼1 eV while their ligand-field splitting can be as large as 7 eV for actinyls. Matsika *et al*.^40^ pointed out that the influence of these effects can be ordered as follows: ax. field (5*f*σ*, 5*f*π*)>spin–orbit>ax. field (5*f*δ, 5*f*ϕ)+eq. field. The ax. field exerts the strongest effect caused by the strong covalent nature of the An-O_ax_ bond. As a result, the 5*f*σ* and 5*f*π* states are shifted with respect to the 5*f*δ and the 5*f*ϕ orbitals (5*f*δ≈5*f*ϕ<5*f*π*<<5*f*σ*). The shift is stronger for the 5*f*σ* than the 5*f*π* states due to interaction of the filled An-O σ_υ_ orbital and the semi-core 6*p*_*z*_ orbital with the same σ_υ_ symmetry (‘pushing from below’)^1^,^41^,^42^,^43^,^44^. It is the formation of a σ_υ_ hybrid orbital from An 5*f* and pseudocore 6*p*, mixed with O_ax_ 2*p* valence orbitals, that is the source of high covalency of the U-O_ax_ bond. The mixing coefficient (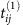) of a metal (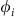) with ligand (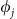) orbitals is associated with the covalency of the bond; it can be large due to their strong overlap or near degeneracy as described to the first order by perturbation theory: 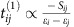, where 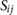 and 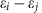 are the overlap and energy difference of the orbitals, respectively^2^,^12^. The change of these two parameters, 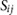 and 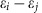, can lead to increase of 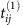 and thereby to increase of overlap or energy-driven covalency^2^,^45^. Denning^3^ pointed out that the strong overlap of the 6*p* with 2*p* orbitals plays a substantial role for the covalency of the UO_2_ ^2+^ bond. Changes in this overlap lead to variations in the filled-filled interaction and is manifested in the HR-XANES spectra as the observed changing peak energy differences, most substantially between the 5*f*δ/ϕ and 5*f*σ* peaks. In Fig. 5, the energy difference between the main 5*f*δ/ϕ peak and the 5*f*π*/5*f*σ* peaks for Np is significantly smaller (Δ5*f*δ/ϕ−5*f*π*=1.6 eV, Δ5*f*δ/ϕ−5*f*σ*=5.4 eV) compared to that for U (Δ5*f*δ/ϕ−5*f*π*=2.3 eV, Δ5*f*δ/ϕ−5*f*σ*=6.4 eV) (Supplementary Fig. 3; Supplementary Table 3). The trend is preserved for Pu but difficult to quantify since the 5*f*δ/ϕ and 5*f*π* peaks are not resolved in the HR-XANES spectrum. Note that the Np 5*f*σ* state lies 1 eV nearer to the 5*f*δ/ϕ orbital than for U, whereas the 5*f*π* is 0.7 eV nearer. Since the 5*f*π* orbital is closer to the nucleus the effect cannot be explained with mere contraction of the 5*f* orbitals. In an *in-situ* experiment, we electrochemically reduced [U(VI)O_2_(CO_3_)_3_]^4−^ to [U(V)O_2_(CO_3_)_3_]^5−^ and observed the same qualitative result; energy differences are much smaller for U(V) (Δ5*f*δ/ϕ−5*f*π*=1 eV, Δ5*f*δ/ϕ−5*f*σ*=3.4 eV) compared to those for U(VI) (Δ5*f*δ/ϕ−5*f*π*=1.6 eV, Δ5*f*δ/ϕ−5*f*σ*=5.1 eV). The additional electron in the U(V) system lowers the covalent character of the U-O_ax_ bond, which is expressed in the smaller energy difference between the 5*f*δ/ϕ and the 5*f*π*/5*f*σ* peaks. The same effect is observed by comparison of the U M_4_ HR-XANES spectra of UO_3_·1–2(H_2_O) (metaschoepite) (Δ5*f*δ/ϕ−5*f*π*=2.1 eV, Δ5*f*δ/ϕ−5*f*σ*=5.6 eV) and CaU_2_O_7_ (Δ5*f*δ/ϕ−5*f*π*=1.7 eV, Δ5*f*δ/ϕ−5*f*σ*=4.2 eV). The U-O_ax_ bond length is elongated (Δ*R*=+0.16 Å) in the latter, which is also associated with decreased covalency of the bond^18^,^42^. We propose to use the relative energy difference between the 5*f*δ/ϕ and the 5*f*π*/5*f*σ* HR-XANES spectral peaks as a qualitative measure of relative changes in the overlap-driven covalency of the actinyl bond^2^.

Extended X-ray absorption fine structure (EXAFS) and calculations with density functional theory (DFT) using relativistic effective core potentials (RECPs) were performed previously for the AnO_2_ ^2+^ system^23^. Going from UO_2_(H_2_O)_5_^2+^ to PuO_2_(H_2_O)_5_^2+^ the An-O_ax_ bond distance slightly decreases (R_U-Oax_=1.76 Å^46^/1.78 Å^47^, R_Np-Oax_=1.75 Å^48^, R_Pu-Oax_=1.74 Å^49^), suggesting strengthening of the chemical bond and more electronic density on the An atom. This has been theoretically studied and the net overlap population of the U 5*f* with the O valence orbitals was found to decrease by about 10% going from U to Pu, explained with the contraction of the 5*f* orbitals across the An series^23^,^40^. More recent experiments and calculations for AnO_2_^+/2+^ support the concept that the covalency of the An increases with the An *Z* number across the actinide series^45^,^50^,^51^. One strong argument is the less favourable oxo-exchange of AnO_2_^+/2+^ with methanol and water going from U to Pu^51^. The theoretical results are inconclusive but prevails the notion that this is an energy-driven covalency. This hypothesis is underpinned by the observed trends for the 5*f* orbitals in our RIXS maps. The stronger localization of the 5*f* orbitals likely leads to a better energy match with the O 2*p* orbitals for Pu, a less optimal energy match for U.

### Calculations of Pu(VI) M_5_ absorption edge HR-XANES spectra

To answer the question if the An M_4,5_ absorption edge HR-XANES spectra are a good approximation of the ground state partial An 5*f* density of state, we have explicitly considered the role of multiplets in the Pu M_4,5_ absorption edge HR-XANES spectrum. The multiplets arise from the angular momentum coupling of the open-shell electrons, both for the core-hole as well as the open 5*f* shell where the excited state occupation of the 5*f* shell must be used. The energy spacing of these multiplets may be reasonably large and this splitting will cause the XANES spectra to differ from a ground state partial density of states (DOS). It is well known that these multiplet effects dominate in Ln M edge spectra^52^. An example of the different importance of multiplets in the XANES of closed shell systems depending on the spin–orbit splitting in the excited core-level is given by Bagus *et al*.^25^. U(VI) does not have 5*f* electrons in the ground state, and we showed previously that the spectrum can be well-described with ground state DFT electronic structure calculations^17^; the deviations between calculated and measured relative energies are <0.6 eV. Hay *et al*. compared splitting of the virtual orbitals for 5*f*^1^ and 5*f*^2^ configurations for PuO_2_^3+^ and PuO_2_ ^2+^, and found a significant spread of orbital energies^23^,^53^. Electron–electron interactions become important in the ground state when a second electron is added (5*f*^2^). Clearly the broadening and the strong overlap of the 5*f*δ/ϕ and the 5*f*π* orbitals in the PuO_2_ ^2+^ M_5_ HR-XANES spectrum is partially caused by such multiplet effects.

**Figure 6:**
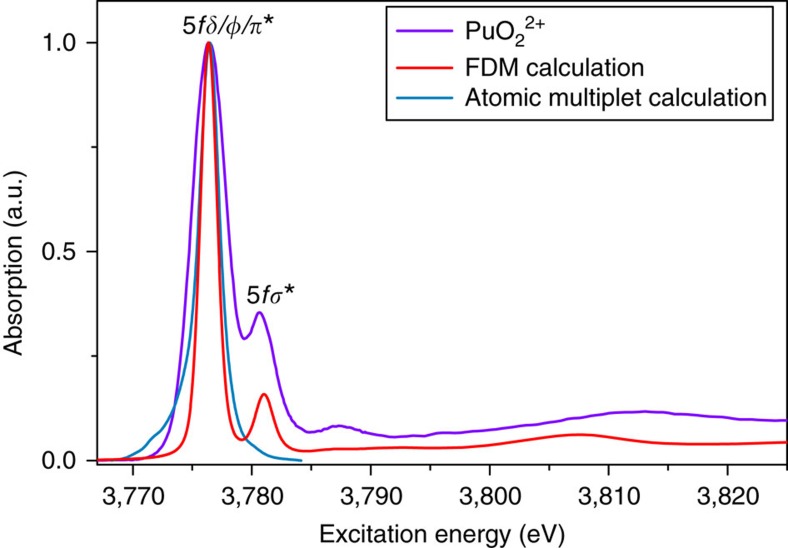
Calculated and experimental Pu M_5_ HR-XANES spectra. Pu M_5_ absorption edge HR-XANES experimental and calculated spectra of PuO_2_ ^2+^ in 1 M HClO_4_, The atomic multiplets are from a full relativistic treatment of initial and final states with the DIRAC code^21^; the FDM calculations are performed with the FDMNES code^22^.

We performed atomic multiplet calculations to examine the effect of multiplet splittings and to compare excited state calculated spectra with our HR-XANES measurement on hydrated PuO_2_ ^2+^ (Supplementary Note 2). The results are depicted in Fig. 6. Only the main peak is reproduced for calculations taking only the atomic multiplets into account. Furthermore, the width of the main peak for the atomic multiplet XANES is significantly narrower than the width measured in the HR-XANES. The theoretical width is obtained with a broadening for resolution and lifetime as determined for the HR-XANES (Supplementary Note 2; Supplementary Fig. 4). The smaller width of the atomic multiplet calculation is an evidence for the presence of unresolved multiplets arising from the ligand-field splittings. This is a clear sign that the ligand field is important for correct theoretical description of the Pu spectrum; similar evidence has been also recently shown for ThO_2_ (ref. 54). For the FDMNES calculation of PuO_2_(H_2_O_5_)(ClO_4_)_2_ spectra based on a single-electron approximation, the satellite features of the main peak are present, although this method does not take electron–electron correlation effects into account (Fig. 6). The best agreement between the calculated FDMNES spectrum and the experiment was achieved for the ground state calculations, that is, no inclusion of screening of the core-hole, approximating electron–hole interactions. The calculated spectrum (Fig. 6) and the partial 5*f*-DOS (Supplementary Note 2; Supplementary Fig. 5) closely resemble the experimental PuO_2_ ^2+^ M_5_ edge HR-XANES up to 3,790 eV. The post edge features in the theoretical spectrum are shifted to lower energies compared to experiment. That no core-hole screening was needed for reproducing the experimental spectra also substantiates the strong localization of the 5*f* states on the Pu atom. There are substantial differences in screening of the core-hole when the electron is in the bound states and when it is in the continuum. We observed these differences manifested in the RIXS map. The calculated intensities somewhat deviate from the experimental spectrum.

## Discussion

We illustrate the unique utility that An 3*d*4*f* RIXS offers in characterizing actinide electronic structures; it allows direct determination and comparison of the level of 5*f* orbital localization and these orbitals’ participation in the chemical bonding for any type of materials, under static or dynamic conditions, not possible with other methods. We have applied this technique to U, Np and Pu in the actinide series and find that the localization of the U and Np 5*f* orbitals varies for different materials, but does not change for Pu, although studied in several different oxidation states (III, IV, VI) and states of matter (solid, solution). This result indicates that the 5*f* states in Pu are less active in the chemical bonding, compared to U and Np, and contrasts the general notion that the 5*f* orbitals are widely responsible for the ability of Pu to co-exist in several different oxidation states^24^. Investigations of additional An materials and quantum-chemical calculations of An 3*d*4*f* RIXS maps will be very beneficial for verification of these conclusions. Covalent mixing between the An 6*d* and the O 2*p* orbitals exists too and it might play an important role^55^,^56^. Further, changes in the relative energy differences between the 5*f*δ/ϕ and the 5*f*π*/5*f*σ* orbitals, determined from their RIXS and HR-XANES, provide a direct qualitative measure for the level of overlap-driven covalency in the actinyl bond. The observed trends in these energy differences indicate that U(VI)-O_ax_ is more covalent compared to the Np(VI)-O_ax_ and Pu(VI)-O_ax_ bonds due to stronger orbital overlap of U 5*f*/6*p* and O 2*p* orbitals. The An M_4,5_ absorption edge HR-XANES and 3*d*4*f* RIXS can help to distinguish between the classical notion of overlap-driven covalency and energy-driven covalency. Apparently the overlap-driven covalent character of the An-O binding in the actinyl cations decrease within the U-Pu series, while the energy-driven covalent character increases without increasing the electron density of the binding. Taking into account, the results on oxygen isotope exchange of Lucena *et al*.^51^ and the computational results of Kaltsoyannis^12^, the energy-driven covalency might have a higher impact on chemical binding stability/strength than the overlap-driven covalency.

The An M_4,5_ absorption edge HR-XANES spectra qualitatively resemble the partial 5*f*-DOS of the An (Supplementary Note 2; Supplementary Fig. 5). It was shown that electron–electron interactions lead to broadening but are not the dominant effect in the An M_4,5_ absorption edge HR-XANES spectra. The axial ligand field plays the most important role and therefore it needs to be considered in calculations.

## Methods

### Preparation of the samples

All preparation procedures of the UO_2_ ^2+^, NpO_2_ ^2+^ and PuO_2_ ^2+^ samples were performed in Ar glove boxes. The UO_2_ ^2+^ and NpO_2_ ^2+^ solutions were prepared by dissolving calculated amounts of Na_2_U_2_O_7(cr)_ and Na_2_Np_2_O_7(cr)_ in 1 M HClO_4_ aqueous solution. The PuO_2_ ^2+^ was prepared starting from aqueous Pu^4+^ in an electrochemical cell equipped with Pt-mesh working, Pt-wire counter and Ag/AgCl reference electrodes. Each sample contained ∼0.06 M U/Np/Pu. The U(VI) and Pu(VI) samples were 100% pure; whereas ultraviolet-vis-NIR spectroscopy performed after the HR-XANES experiments indicated the Np(VI) sample to contain <25 % Np(V). Overall, 135 μl aliquot of each sample was measured in specially designed liquid cells made of polyether ether ketone (PEEK) equipped with 10 μm thick Kapton windows. These cells comprised the first containment and were placed in a second containment inert gas cell prior transportation to the beamline, mounting and measurement (Supplementary Note 1; Supplementary Table 2).

### Experiments

The U M_4_ (3,728 eV), Np M_5_ (3,664 eV) and Pu M_5_ (3,775 eV) edge^57^ HR-XANES/RIXS spectra of UO_2_ ^2+^, NpO_2_ ^2+^ and PuO_2_ ^2+^ were measured using the Johann type X-ray emission spectrometer installed at the INE-Beamline for actinide research at the ANKA synchrotron radiation facility, Karlsruhe, Germany^14^,^58^. The incident energy was monochromatized by a Si(111) double crystal monochromator (DCM). Sample, five analyzer crystals and a single diode VITUS silicon drift detector (VITUS SDD KETEK) were arranged in a vertical Rowland geometry^59^; A box encompassing the spectrometer and maintaining constant He flow was installed to avoid intensity losses due to scattering and absorption of photons. The HR-XANES spectra were obtained by recording the maximum intensity of the M_α_/M_β_ emission lines (U M_β_, Np M_α_, Pu M_α_) diffracted by the five spherically bent Si(220) crystal analyzers (SAINT-GOBAIN) with 1 m bending radius and focused onto a single diode VITUS SDD (Supplementary Table 4). The crystals were aligned at 75.17° (U), 83.42° (Np) or 75.22° (Pu) Bragg angles. The RIXS maps of the UO_2_ ^2+^, spent nuclear fuel (SNF, UO_2_), high-burn up structure of SNF (HBS, UO_2_)^60^,^61^,^62^, [UO_2_(CO_3_)_3_]^5−^, [UO_2_(CO_3_)_3_]^4−^, (ref. 63) [UO_2_(H_2_O)_5_](ClO_4_)_2(cr)_, NpO_2(am,hyd)_, Ca_0.5_NpO_2_(OH)_2_·1.3H_2_O, Na_2_Np_2_O_7(cr)_ (refs 64, 65), NpO_2_ ^2+^, PuO_2(cr)_ (ref. 66), Pu(III) (ref. 67), Pu(IV) and PuO_2_ ^2+^ were recorded by measuring HR-XANES spectra across a M_α_/M_β_ normal emission line. The DCM was calibrated by assigning 3,725.2 and 3,775 eV to the maxima of the WLs of the U/Pu M_4_/M_5_ edge HR-XANES spectra of a UO_2_ and a PuO_2_ samples, respectively. The experimental energy resolution was 1.2 eV (U/Pu) or 1 eV (Np), estimated by measuring the full width at half maximum of the elastically scattered incident beam (Supplementary Tables 1 and 4).

### Multiplet calculations

An isolated Pu^6+^ cation served as a model for our fully relativistic calculations of the wavefunctions for the analysis of the Pu M_5_ HR-XANES.

Wavefunctions (WFs) were determined with a Dirac–Hartree–Fock calculation of four-component spinors followed by a complete open-shell configuration interaction, COSCI, to treat the angular momentum coupling of the open-shell electrons. The Dirac–Coulomb Hamiltonian without Breit and higher order interactions was used; furthermore an approximation for a class of two-electron integrals involving the small components was also used. These approximations have been tested and found to lead to only small changes. These WFs treat scalar relativistic effects, spin–orbit splittings, and the angular momentum coupling of the open-shell electrons on an equal footing.

The approach is completely non-empirical and no parameters in the calculation of the WFs are adjusted to fit experiment. The application of these methods to core-level ionizations, X-ray photoemission spectroscopy or XPS, has been reviewed in ref. 55 and references therein. Calculations of the WFs were performed with the DIRAC programme^21^ and calculations of the *I*_rel_ were performed with the CLIPS programme^68^. Large flexible basis sets were used to expand the orbitals. For Pu, the basis set was taken from the Dyall basis sets distributed with DIRAC^21^ and for O, the same basis set was used as in our earlier work on heavy metal oxides (Supplementary Note 2)^55^,^69^.

### FDMNES calculations

FDMNES^22^ is an *ab initio* code simulating the spectroscopies related to the X-ray absorption process. It contains a time-dependent DFT extension, but this study was performed using the standard DFT approach. The electronic structure can be calculated using the multiple scattering theory under the muffin-tin approximation on the potential shape, but in the present study better results are obtained in its more precise scheme using the finite difference method^70^, which allows a free-shape potential. To get the absorption cross section, FDMNES first solves the electronic structure in the cluster probed by the photoelectron around the absorbing atom, giving thus the projected DOS in the atoms. This calculation can be, on demand, self-consistent or not, relativistic, with or without spin–orbit. In this last case, we follow Wood and Boring^71^ solving explicitly only the large component orbital instead of the four determined in the Dirac–Slater calculations. This reformulation gives a couple of Schrödinger-like equations closely akin but improved to the Pauli equation. Different imperfect schemes can be used to improve the excited state description by the taking into account of the core-hole with different model of screening. Nevertheless, we found here that the best choice was the simulation in the ground state. The code benefited from recent improvements by Guda *et al*.^72^ in the numerical process, allowing simulations on PC, here with a 3.5 Å radius cluster containing 18 atoms, without any symmetry, but identity, and with spin–orbit. The calculations shown in Fig. 6 and Supplementary Fig. 5 are self-consistent, relativistic and include spin–orbit coupling (Supplementary Note 2).

### Data availability

The data can be provided by the authors on request.

## Additional information

**How to cite this article:** Vitova, T. *et al*. The role of the 5*f* valence orbitals of early actinides in chemical bonding. *Nat. Commun.* **8,** 16053 doi: 10.1038/ncomms16053 (2017).

**Publisher’s note:** Springer Nature remains neutral with regard to jurisdictional claims in published maps and institutional affiliations.

## Supplementary Material

Supplementary Information
