# Supplementary material for: The role of the 5f valence orbitals of early actinides in chemical bonding
Source: Nat Commun. 2017 Jul 6;8:16053. doi: 10.1038/ncomms16053 (PMC5504295; doi:10.1038/ncomms16053)
Supplement: Supplementary Information [file ncomms16053-s1.pdf]

Title of file for HTML: Supplementary Information
Description: Supplementary Figures, Supplementary Tables, Supplementary Notes and Supplementary References

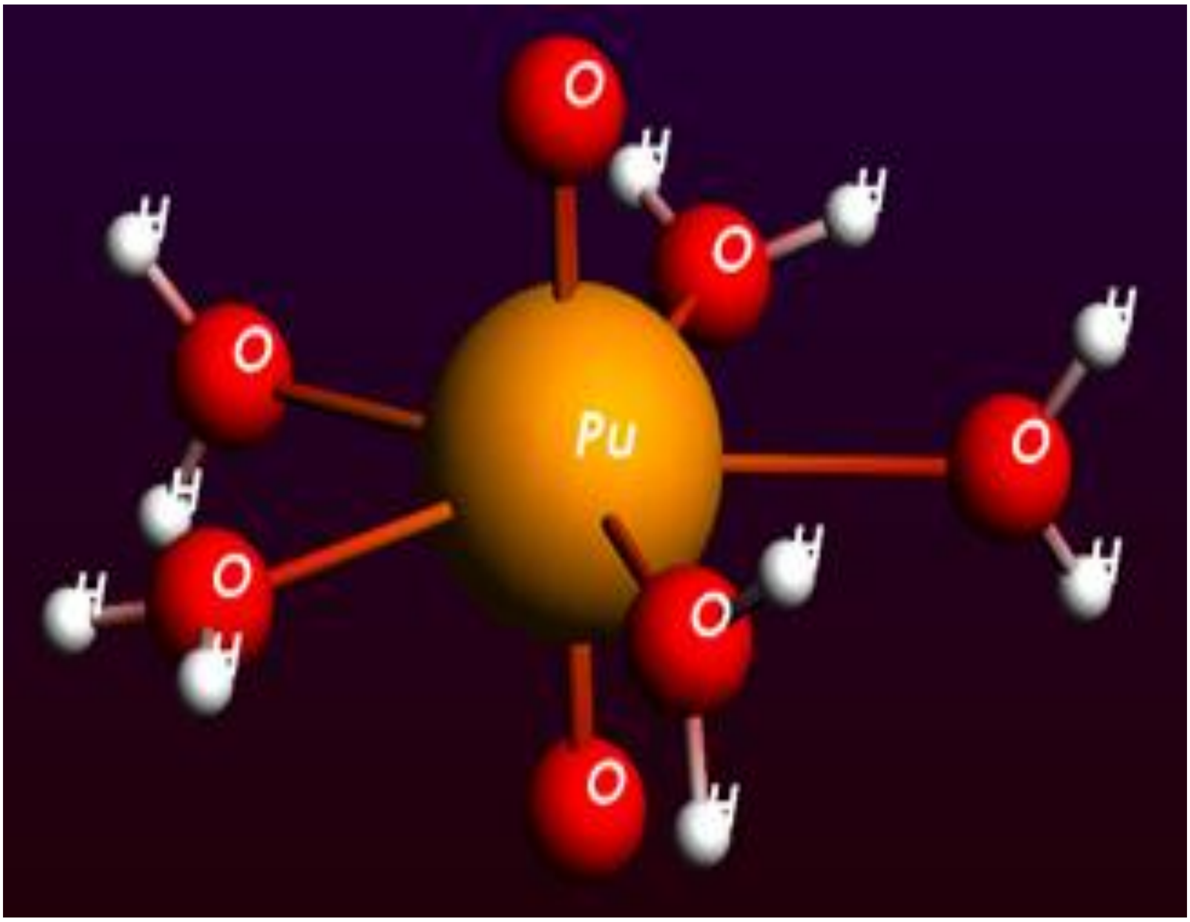

Supplementary Figure 1. PuO_2^{2+} in aqueous solution. A model of PuO_2^{2+} coordinated by five H_2O molecules in the equatorial plane. This $PuO_2(H_2O)_5(ClO_4)_2$ crystalline compound is reported in ICSD number 421144.

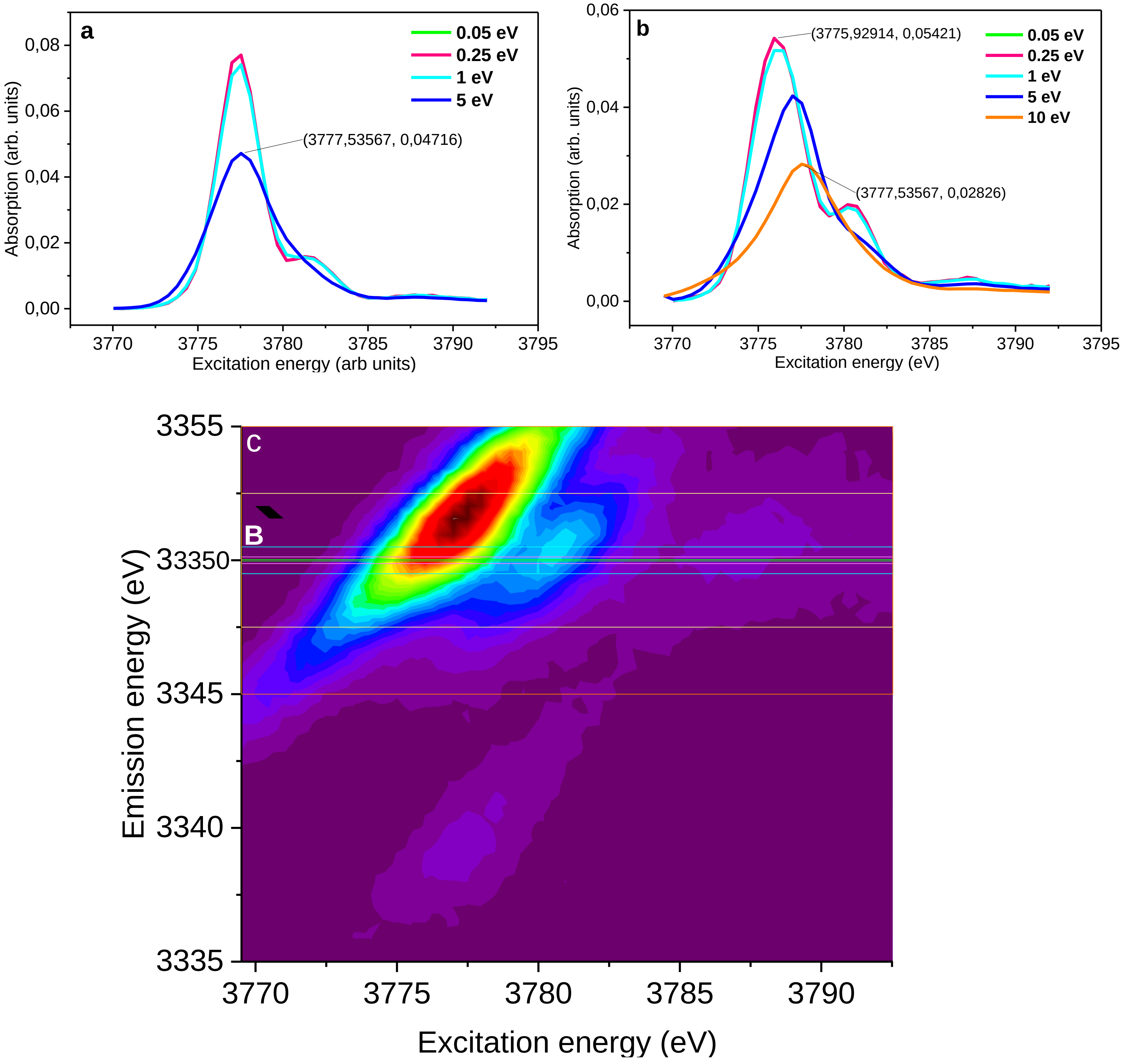

Supplementary Figure 2. Pu M$_5$ edge HR-XANES/XANES and 3*d*4*f* RIXS spectra of PuO_2^{2+}. (**a-b**) Pu M$_5$ edge HR-XANES/XANES spectra of PuO_2^{2+} obtained by integrating regions with variable emission energy widths (green: 0.05 eV, pink: 0.25 eV, cyan: 1 eV, blue: 5 eV, orange: 10 eV) across the 3*d*4*f* RIXS map of PuO_2^{2+}. These are line A (3351.6 eV) (a) and line B (3350 eV) (b) centered regions; (**c**) as an example the line B centered regions of the 3*d*4*f* RIXS map. The XANES spectrum in (**b**) obtained integrating an region with emission energy width of 10 eV corresponds to a Pu M$_5$ edge XANES spectrum measured in conventional fluorescence mode.

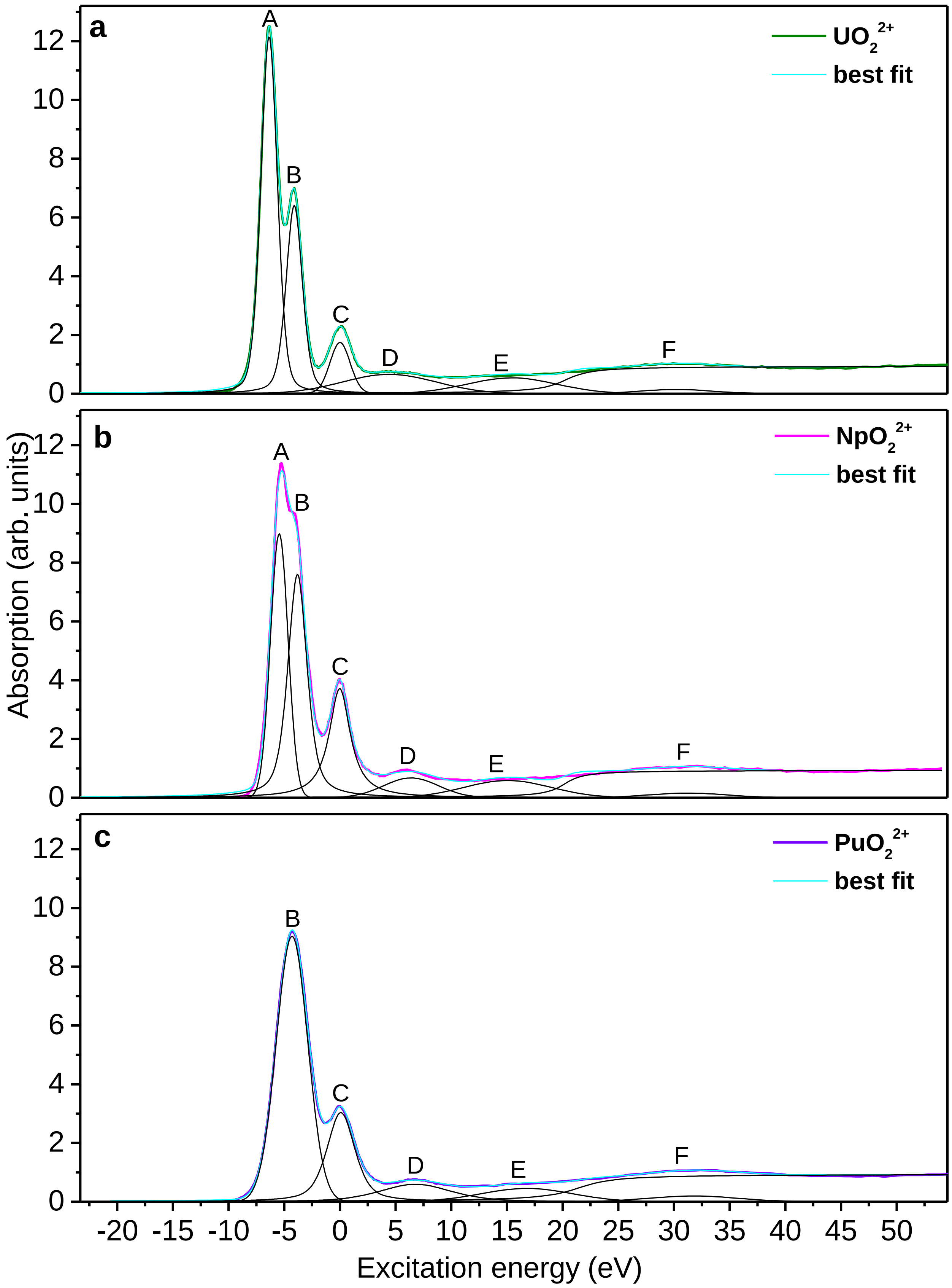

Supplementary Figure 3. Experimental and best fit U M_4 and Np/Pu M_5 edge HR-XANES. The U M_4 and Np/Pu M_5 edge HR-XANES spectra of (**a**) UO_2^{2+},(**b**) NpO_2^{2+} and (**c**) PuO_2^{2+}, their best fits and the Voigt profiles used to model the spectra (black lines).

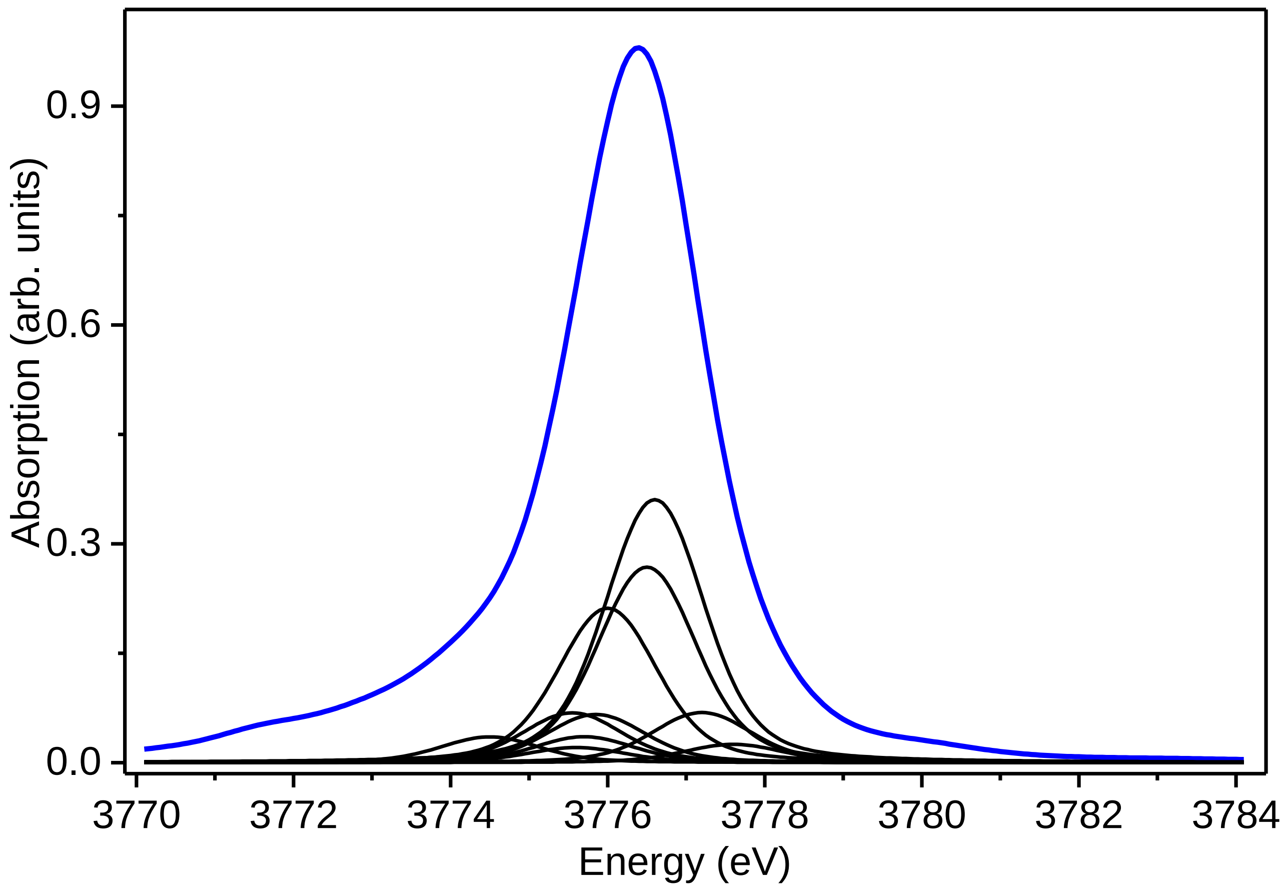

Supplementary Figure 4. Calculated Pu M_5 edge HR-XANES of Pu(VI). Pu M_5 edge HR-XANES atomic multiplet calculation of Pu(VI) including individual transitions. The atomic multiplets are from a full relativistic treatment of initial and final states with the DIRAC code [1]

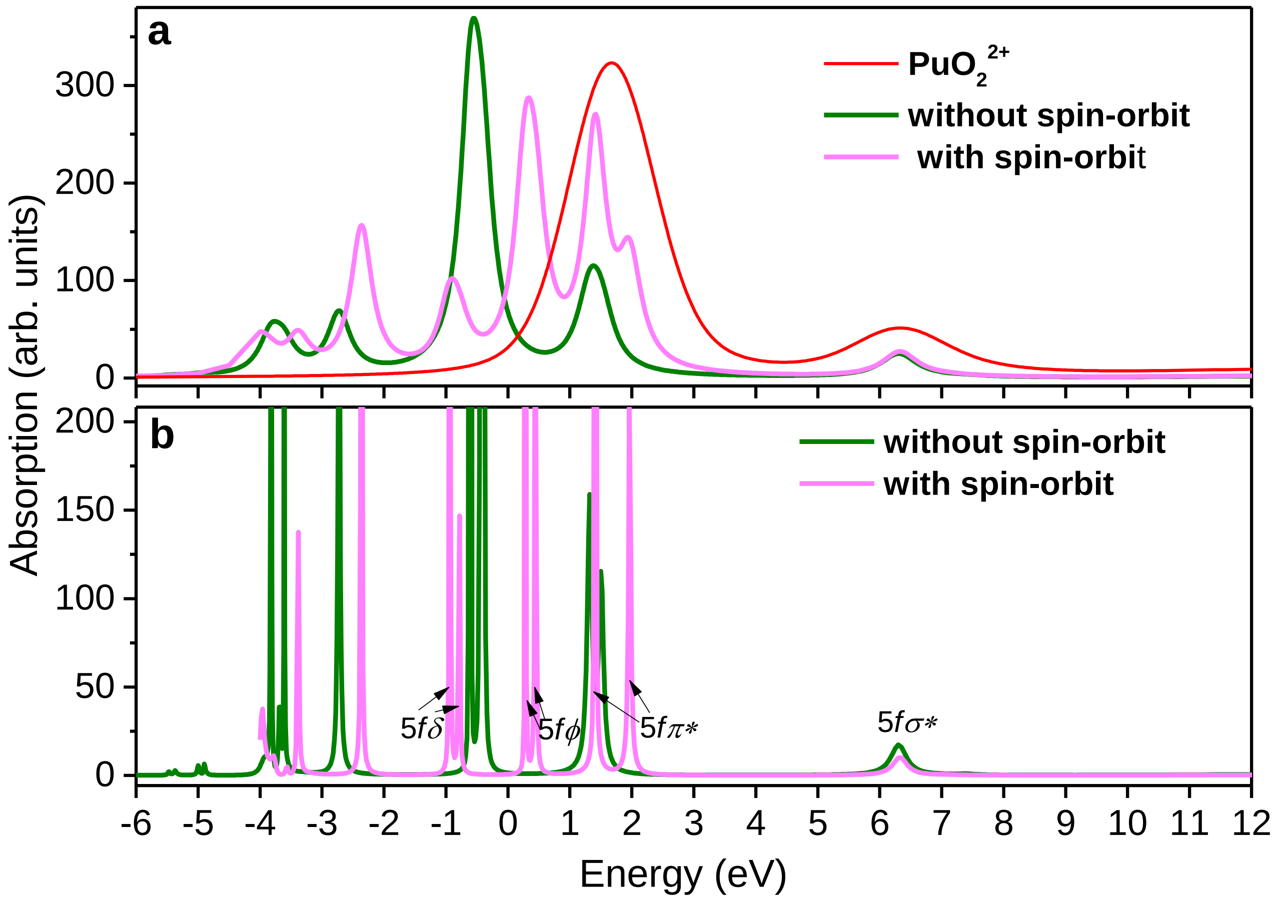

Supplementary Figure 5. Calculated Pu M_5 edge HR-XANES and DOS of Pu(VI). (**a**) Convoluted (with spin-orbit) and non-convoluted Pu M_5 HR-XANES of PuO_2^{2+} and (**b**) Pu 5*f* density of states (DOS) spectra with and without spin-orbit coupling included in the finite difference method (FDM) calculations. The Fermi energy position is at +1.41 eV and it divides the occupied from the unoccupied molecular orbitals (MO). The unoccupied MO are marked in (**b**) for the calculation "with spin orbit".

Supplementary Table 1. Summary of parameters. The table lists: studied element (**Z**); measured absorption edge (**Edge**); measured fluorescence line (Line); analyzer crystals (**Crystal**); Bragg angle (**Angle**); calculated experimental energy resolution (**E_{tot}** calc.), full width at half maximum of the elastically scattered radiation as a measure of the experimental energy resolution (**E_{tot}** meas.); core-hole lifetime broadening of the intermediate (**Γ_{3d}**) and final (**Γ_{4f}**) states; core-hole lifetime broadening contributing to the spectrum calculated as **Γ** = $\frac{1}{\sqrt{(1/\Gamma_{3d})^2+(1/\Gamma_{4f})^2}}$ (**Γ**)^2; calculated total energy resolution (**Γ_{tot}**)^2.

**Z**	**Edge (eV)**	**Line (eV)**	**Crystal**	**Angle**	**E_{tot} meas. (eV)**	**E_{tot} calc. (eV)**	**Γ_{3d} (eV)**	**Γ_{4f} (eV)**	**Γ^2 (eV)**	**Γ_{tot}^2 (eV)**
U	M_4: 3728	Mβ: 3339.8	Si(220)	75.17°	1.2	0.9	3.2[3]	0.28[4]	0.3	1.2
Np	M_5: 3664	Mα: 3250	Si(220)	83.42°	1	0.7	3.3*	0.4*	0.4	1.0
Pu	M_5: 3775	Mα: 3339	Si(220)	75.22°	1.2	0.9	3.4*	0.5*	0.5	1.3

*The value is estimated

Supplementary Table 2. Sample list. List with samples studied with the An $M_{4,5}$ HR-XANES and 3*d*4*f* RIXS. Techniques

	**Sample**
1	U(IV)_1: UO_2 in spent nuclear fuel (SNF)
2	U(V): $[UO_2(CO_3)_3]^{5-}$
3	U(IV)_2: UO_2 in high burn up structure (HBS)
4	U(VI)_3: $[UO_2(CO_3)_3]^{4-}$
5	U(VI)_1: UO_2^{2+} in 1M $HClO_4$
6	U(VI)_2: $[UO_2(H_2O)_5](ClO_4)_{2(cr)}$
7	Np(VI)_2: $Na_2Np_2O_{7(cr)}$,
8	Np(V): $Ca_{0.5}NpO_2(OH)_2 \cdot 1.3H_2O$,
9	Np(IV): $NpO_{2(am,hyd)}$,
10	Np(VI)_1: NpO_2^{2+} in 1M $HClO_4$
11	Pu(III): Pu in 1M $HClO_4$
12	Pu(IV)_1: Pu in 1M $HClO_4$
13	Pu(IV)_2: $PuO_{2(cr)}$
14	Pu(VI): PuO_2^{2+} in 1M $HClO_4$

Supplementary Table 3. Fit parameters. An element (**Z**) and absorption feature (**Feature**), height (**Height**), position (**Position**), and full width at half maximum (**Fwhm**) parameters of the pseudo Voigt (**PV**) functions used to model the U M_4 and Np, Pu M_5 edge HR-XANES spectra and an arctangent used to model the edge jump, the gauss part (**α**) and the area (**Area**) of the PV profiles, and the residual between experimental data and best fit.

**Z**	**Feature**	**Height ± 0.01**	**Position ± 0.1 (eV)**	**Fwhm ± 0.1 (eV)**	**α**	**Area**	**Residual (%)**
U	A	12.16	-6.4	1.8	0.8	25.1	2.1
Np	A	8.96	-5.4	1.9	1	17.7	4.1
Pu	A						
U	B	6.39	-4.1	1.7	0.7	13.5	2.1
Np	B	7.6	-3.8	2.0	0.5	20.5	4.1
Pu	B	9.03	-4.3	3.3	1	31.9	1.5
U	C	1.72	0.0	2.1	1	3.9	2.1
Np	C	3.71	0.0	2.3	0.05	12.8	4.1
Pu	C	3.03	0.1	3.0	0.5	11.7	1.5
U	D	0.67	4.4	10.4	1	7.4	2.1
Np	D	0.69	6.5	6.0	1	4.5	4.1
Pu	D	0.60	6.8	7.6	0.6	5.6	1.5
U	E	0.54	15.7	9.2	1	5.3	2.1
Np	E	0.59	15.1	8.6	1	5.4	4.1
Pu	E	0.45	16.8	9.6	1	4.6	1.5
U	F	0.14	30.2	7.7	1	1.2	2.1
Np	F	0.15	31.2	8.4	1	1.4	4.1
Pu	F	0.19	31.7	9.3	1	1.9	1.5
U	Arctan	0.94 [a]	20.3	5.5			2.1
Np	Arctan	0.94 [a]	19.9	3.8			4.1
Pu	Arctan	0.94	21.4	7.7			1.5

Supplementary Tables 4. **Experimental parameters.** The table lists: name of sample (**Sample**); Energy regions used for the measurements of the RIXS (**RIXS**) and HR-XANES (**HR-XANES**) spectra of the UO_2^{2+}, NpO_2^{2+} and PuO_2^{2+} samples. Two spectra are measured for each sample.

**Sample**	**RIXS**	**HR-XANES**<br>All regions are relative to the maximum of the first derivative of the HR-XANES spectra
UO_2^{2+}	Excitation energy 3.72 - 3.74 keV, step size: 0.2 eV,<br>integration time 1 s<br>Emission energy 3.327 - 3.338 keV, 0.33 eV, 1 s	-15 - -5 eV, step-size 0.5 eV, integration time 1 s<br>-5 - +15, 0.1, 1<br>+15 – +65, 0.5, 1
NpO_2^{2+}	3.659 - 3.684, 0.5, 2<br>3.248 - 3.268, 0.25, 2	-15 - -5 eV, step-size 0.5 eV, integration time 1 s<br>-5 - +15, 0.1, 1<br>+15 – +65, 0.5, 1
PuO_2^{2+}	3.77 - 3.793, 0.5, 3<br>3.335 - 3.355, 0.5, 3	-15 - -5 eV, step-size 0.5 eV, integration time 1 s<br>-5 - +15, 0.1, 1<br>+15 – +65, 0.5, 1

Supplementary Note 1. Samples.

UO_2 in spent nuclear fuel (SNF, U(IV)_1) and high burn-up structure (HBS, U(IV)_2). The studied SNF samples were taken from the SBS1108 N0204 fuel rod segment, which was irradiated in the pressurized water reactor Gösgen nuclear power plant in Switzerland. The irradiation was carried out in four cycles for a period of time of 1226 days with an average linear power rate of 260 W/cm and achieving an average burn-up of 50.4 GWd/t_{HM}. The fuel rod segment was discharged the 27th of May 1989 that implies a cooling time of 24 years before characterization and cutting of the segment. Characteristic data of the studied SBS1108 N0204 fuel rod segment are given in.[5,6] A pellet size sample (about 10 mm length) was cut in the gap between two adjacent pellet of the fuel rod segment. The pellet was mechanically decladded and fractured. The high burn-up structure[7] sample (HBS, U(IV)_2) was prepared by fixing the SNF dust stuck on the cladding wall of the emptied pellet on a Kapton tape strip. The dust particles size well below 300 µm and belong to the outer region of the SNF pellet, where the HBS is formed. The second SNF sample (U(IV)_1) has been prepared by bringing a particle of several mm size, which was obtained from the centre region of a the fractured SNF pellet, in contact with a Kapton tape. The on the glue caught SNF footprint particles size about 1 mm. Both samples have then be covered with 13 µm thick Kapton foil and mounted in a Plexiglas cell with an additional 8 µm Kapton foil containment for measurement. The HBS U(IV)_2 sample is partially oxidized to U_4O_9, which contains U(IV) and U(V), due to the small particle size.

Electrochemical preparation of $[UO_2(CO_3)_3]^{5-}$ (U(V)) and $[UO_2(CO_3)_3]^{4-}$ (U(VI)_3) in 1M Na_2CO_3. The electrochemical preparation and the recording of the UV-Vis spectra and the cyclic voltamogramms of the samples were first performed in an Ar glove box following the description of Ikeda et al.[8] The same cell was used for the *in-situ* spectroelectrochemical experiments at the INE-Beamline. This cell enables coupling of UV-Vis spectroscopy with electrochemistry and X-ray absorption/emission spectroscopy.[9] A Pt-mesh (80 µm, 25×35 mm) was used as a working electrode, a Pt-spiral (0.5 mm Ø, 23 cm length) as a counter electrode and Hg/HgO as a reference electrode (ALS Co., Ltd). The negative potential of -775 mV determined from cyclic voltammetry measurements was applied to reduce U(VI) to U(V).[8] UV-Vis spectra were continuously recorded during the electrochemical reaction. The intensity of the U(VI)-yl characteristic UV-VIS peaks diminished after 120 minutes of electrolysis changing the color of the solution from yellow to colorless. These were clear evidences for complete reduction of U(VI) to U(V). The U(V) species remained stable for at least 800 minutes under anoxic conditions (flushed with He continuously. The RIXS/HR-XANES spectra were measured prior and after the U(VI) reduction was completed.

Electrochemical preparation of Pu(III), Pu(IV) (Pu(IV)_1) and Pu(VI) in 1M $HClO_4$. The sample preparations were carried out in an Ar glove box. Aliquot of purified colloid free 0.06 M Pu(III/IV) stock solution in 1 M $HClO_4$ was quantitatively reduced to Pu(III) at a potential of E = -400 mV [all E values are measured versus Ag/AgCl + 3 M NaCl].[10] Rapid oxidation of Pu(III) at E = 900 mV yielded a solution of 91 % Pu(IV)aq / 6 % Pu(VI)aq. Subsequent oxidation at E = 1900 mV led to pure Pu(VI) solutions. 100 % purity was achieved for the Pu(III) and Pu(VI) solutions. The Pu(IV) in 1M $HClO_4$ samples contained 6 % of Pu(VI). Vis-NIR spectra of the Pu samples were measured prior and after the RIXS/HR-XANES. For the RIXS/HR-XANES experiments the liquid samples were placed in polyether ether ketone (PEEK) cells with 13 µm thick Kapton windows. These cells were placed in an inert gas cell

with Kapton windows with 8 µm thickness, which has an adapted design of the standard INE-Beamline inert-gas cell,[11] including larger windows and inner volume.

Solid samples $NpO_{2(am,hyd)}$ (Np(IV)), $Ca_{0.5}NpO_2(OH)_2 \cdot 1.3H_2O$ (Np(V)), $Na_2Np_2O_{7(cr)}$ (Np(VI)_2), PuO_2 (Pu(IV)_2) and $[UO_2(H_2O)_5](ClO_4)_{2(cr)}$ (U(VI)_2). The preparations and characterizations of the $NpO_{2(am,hyd)}$, $Ca_{0.5}NpO_2(OH)_2 \cdot 1.3H_2O$, $Na_2Np_2O_{7(cr)}$ and the PuO_2 samples are described in D. Fellhauer et. al[12,13] and D. Prieur et. al[14], respectively. 1 mg PuO_2 was mixed with 20 mg BN. 2 mg of Np containing suspension was used for each Np sample. The Np/Pu samples were placed in the inner parts of Al cells. The inner and outer compartments of the cells were covered with Kapton windows with 13 and 8 µm thick Kapton films, respectively.

For the preparation of $[UO_2(H_2O)_5](ClO_4)_{2(cr)}$ a solution of $UO_2(NO_3)_2 \cdot 6H_2O$ (Merck) in 0.1 M perchloric acid was slowly evaporated to near dryness by heating. The precipitate was re-dissolved in 10 ml 0.1 M perchloric acid and the clear solution was again evaporated to near dryness by heating. This procedure was repeated twice. The final solution contained U(IV) in dilute perchloric acid. This solution was concentrated by slow evaporation and let aside. Yellow crystals having the composition $UO_2(ClO_4)_2 \cdot 5H_2O$ were obtained from this solution. These crystals were found to be very hygroscopic.

Supplementary Note 2. Modeling and computational details.

Modeling of HR-XANES. Linear combination least-squares (LCLS) fit analyses of the $M_{4,5}$ edge HR-XANES spectra were performed with the WINXAS program (www.winxas.de) using five pseudo-Voigt (PV) [f(x) = αGaussian + (1 − α)Lorentzian] and one arctangent functions. The Levenberg−Marquardt least-squares algorithm is used in the fit. The results are reported in Supplementary Figure 3 and Supplementary Table 3.

Multiplet calculations. We briefly describe the essential features of our approach. The wavefunctions are built from 4 component spinors, described in a non-relativistic nomenclature as orbitals. These orbitals are solutions of the Dirac Hartree-Fock, DHF, self-consistent field equations. The orbitals are optimized for two separate configurations. First for the ground state configuration, where the M_5 shell is filled and the 5f shell contains 2 electrons. For this configuration, the orbitals are optimized for the average of configurations[15] where the 2 electrons are distributed in all possible ways over the 14 5f orbitals. Second for the excited state configuration, where the M_5 shell contains only 5 electrons and the 5f shell contains 3 electrons; here also the orbitals are optimized for the average of configurations. It is necessary to use two sets of orbitals in order to take into account the screening of the core, M_5, hole by the other electrons, especially those in the shells spatially more extended than the M_5 shell. A special advantage of this approach is that a single set of orbitals can be used to describe the multiplets that arise for these open shell configurations. The angular momentum coupling is determined by mixing the determinants where the open shell electrons are distributed in all possible ways over the $3d_{5/2}$ and 5f shells. The only constraint is that the number of electrons in each shell is fixed as for the configuration used to optimize the orbitals. These are described as configuration interaction, CI, wavefunctions [16] and give reasonably accurate descriptions of the multiplet WF's and energies. It is appropriate to stress that it is not possible to correctly describe multiplets, except in very special cases, with only one determinant; a many determinantly description is essential.
In order to compute the intensities to the different M_5 edge multiplets, we compute the many electron dipole matrix elements between each pair of initial state and final state wavefunctions. Because it is necessary to use different sets of orbitals for the initial and final states, these many electron matrix elements cannot be written in terms of a single reduced matrix element. Rather it is necessary to take suitable sums over products of orbital overlap integrals with orbital dipole moment integrals. The relative intensity, I_{rel}, is taken as proportional to the square of the dipole matrix element where the term in the transition energy, ΔE^3, is neglected since this term is essentially constant over the ~50 eV range of energies of interest given the 3800 eV excitation energy from M_5 to 5f. The transitions are summed over all degenerate intial states of the J=4 ground state level of Pu^{6+} and averaged over all directions, x, y, and z. Our calculated I_{rel} to the discrete states are broadened with a Voigt convolution of a Gaussian, to represent resolution, with an FWHM of 1.2 eV and a Lorentzian, to represent lifetime, of 0.5 eV. These choices are based on the experimental parameters for the HR-XANES measurements. In Figure S4, we show the broadened calculated XANES intensity for the M_5 edge of Pu^{6+}. The black solid line shows the sum over all transitions to excited states while the individual blue solid lines show the largest contributions from the excitations to the individual final multiplets. Clearly, the individual multiplets cannot be resolved.

FDMNES calculations. The calculated Pu M_5 edge HR-XANES spectrum of PuO_2^{2+} is broadened with 1.2 eV Gaussian and 0.5 Lorentzian broadening to simulate the experimental and core-hole lifetime broadening affecting the spectrum.

Input files used for the FDM calculations of the Pu M_5 HR-XANES spectrum of PuO_2^{2+} performed with the FDMNES code.

```
! Indata file for FDMNES, Pu(VI)_HClO4
 Filout
Sim/Pu/Pu_VI/Pu_HClO4_VI_M5_FDM_Rs35_so_SCF
 Range
-4 0.02 4 0.05 10 0.2 15 1 40 2 70
 Quadrupole
 SCF
 Relativiste
 Spinorbite
 Density
 Edge
 M5
 Radius
  3.5
 Z_absorber
  94
 Cif_file
Sim/Pu/in/Pu(VI)_HClO4.cif
 End
```

```
! Indata file for FDMNES - Convolution step
Calculation
Sim/Pu/Pu_VI/Pu_HClO4_VI_M5_FDM_Rs35_so_SCF
EFermi
1.41
Gamma_max
 3
Gamma_hole
 1.5
Gaussian
 1.0
Conv_out
Sim/Pu/Pu_VI/Pu_HClO4_VI_M5_FDM_Rs35_so_SCF_EF141_GM3_GH15_Ga10_conv.txt
 Convolution
 Estart
-20
 End
```

Supplementary References

1. DIRAC. *a relativistic ab initio electronic structure program, Release DIRAC08 (2008), written by L. Visscher, H. J. Aa. Jensen, and T. Saue, with new contributions from R. Bast, S. Dubillard, K. G. Dyall, U. Ekström, E. Eliav, T. Fleig, A. S. P. Gomes, T. U. Helgaker, J. Henriksson, M. Iliaš, Ch. R. Jacob, S. Knecht, P. Norman, J. Olsen, M. Pernpointner, K. Ruud, P. Sałek, and J. Sikkema (see the URL at http://dirac.chem.sdu.dk.* , (2008).
2. de Groot, F.M.F., Krisch, M.H. & Vogel, J. Spectral sharpening of the Pt \textit{L} edges by high-resolution x-ray emission. *Physical Review B* **66**, 195112 (2002).
3. Raboud, P.A., Dousse, J.C., Hoszowska, J. & Savoy, I. ${L}_{1}$ to ${N}_{5}$ atomic level widths of thorium and uranium as inferred from measurements of \textit{L} and \textit{M} x-ray spectra. *Physical Review A* **61**, 012507 (1999).
4. Fuggle, J.C. & Alvarado, S.F. Core-level lifetimes as determined by x-ray photoelectron spectroscopy measurements. *Physical Review A* **22**, 1615-1624 (1980).
5. González-Robles, E. et al. Physico-chemical characterization of a spent UO2 fuel with respect to its stability under final disposal conditions. *MRS Proceedings* **1665**, 283-289 (2014).
6. González-Robles, E. et al. Determination of fission gas release of spent nuclear fuel in puncturing test and in leaching experiments under anoxic conditions. *Journal of Nuclear Materials* **479**, 67-75 (2016).
7. Rondinella, V.V. & Wiss, T. The high burn-up structure in nuclear fuel. *Materials Today* **13**, 24-32 (2010).
8. Ikeda, A. et al. Comparative Study of Uranyl(VI) and -(V) Carbonato Complexes in an Aqueous Solution. *Inorganic Chemistry* **46**, 4212-4219 (2007).
9. Pidchenko, I., Green, J.C., Fellhauer, D., Bahl, S., Prüßmann, T., Bohnert, E., Rothe, J., Geckeis, H., Vitova, T.,. Decrease of covalency in UO_2^+ *in preparation*.
10. Kirsch, R. et al. Oxidation State and Local Structure of Plutonium Reacted with Magnetite, Mackinawite, and Chukanovite. *Environmental Science & Technology* **45**, 7267-7274 (2011).
11. Rothe, J. et al. The INE-Beamline for actinide science at ANKA. *Review of Scientific Instruments* **83**(2012).
12. Fellhauer, D., Neck, V., Altmaier, M., Lützenkirchen, J. & Fanghänel, T. Solubility of tetravalent actinides in alkaline CaCl2 solutions and formation of Ca4 [An (OH) 8] 4+ complexes: A study of Np (IV) and Pu (IV) under reducing conditions and the systematic trend in the An (IV) series. *Radiochimica Acta International journal for chemical aspects of nuclear science and technology* **98**, 541-548 (2010).
13. Fellhauer, D. et al. Np (V) solubility, speciation and solid phase formation in alkaline CaCl2 solutions. Part I: Experimental results. *Radiochimica Acta* **104**, 355-379 (2016).
14. Prieur, D., Carvajal-Nunez, U., Vitova, T. & Somers, J. Local and Electronic Structure of Americium-Bearing PuO2. *European Journal of Inorganic Chemistry*, 1518-1524 (2013).
15. Visscher, L., Visser, O., Aerts, P.J.C., Merenga, H. & Nieuwpoort, W.C. Relativistic quantum chemistry: The MOLFDIR program package. *Computer Physics Communications* **81**, 120-144 (1994).
16. Bagus, P.S., Ilton, E.S. & Nelin, C.J. The Interpretation of XPS Spectra: Insights Into Materials Properties. *Surface Science Reports* **68**, 273 (2013).
